# Could an Innovative Training Program Including Contact Sports and Counseling Help Young People With Traits of Psychopathy and A History of School Dropout?

**DOI:** 10.2174/1745017901915010049

**Published:** 2019-03-26

**Authors:** Federica Sancassiani, Maria Efisia Lecca, Elisa Pintus, Maria Francesca Moro, Roberto Caria, Luigi Minerba, Quirico Mela, Antonio Egidio Nardi, Sergio Machado, Ernesto d’Aloja, Antonio Preti, Mauro Giovanni Carta

**Affiliations:** 1 Department of Public Health and Clinical and Molecular Medicine, University of Cagliari, Cagliari, Italy; 2 Mailman School of Public Health Columbia University, NY, US; 3 Laboratory of Panic and Respiration, Institute of Psychiatry of Federal University of Rio de Janeiro, Rio de Janeiro - RJ, Brazil; 4 Laboratory of Physical Activity Neuroscience, Physical Activity Sciences Postgraduate Program, Salgado de Oliveira University, Niterói - RJ, Brazil

**Keywords:** School dropout, Psychopathic traits, Contact sports, Psychological counseling, Quality of life, (PCL-R)

## Abstract

**Background:**

The aim was to assess the effects of a training program inclusive of contact sports and counseling on school dropout, quality of life (QoL) and psychopathologic symptoms in the youth with a history of school dropout and psychopathic personality traits.

**Methods:**

The Experimental Group (EG) consisted of 32 subjects (male 90.6%; age 19.6±4.3 years); the Control Group (CG) consisted of an equal number matched for gender and age with the same psychological features. At the beginning of the experimental Training Program (T0), both cohorts were assessed by a diagnostic psychiatric interview (SCID ANTAS), the Short Form Health Survey (SF-12) to evaluate QoL, the Psychopathy Checklist - Revised (PCL-R) for the assessment of psychopathic traits, the Self Reporting Questionnaire (SRQ) to measure general psychopathology. At the end of the program (T1), the coorths were evaluated by SF-12 and SRQ.

**Results:**

Twenty-seven subjects in the EG (84.4%) completed the course and underwent the evaluation at T1. The SF-12 score significantly increased from T0 to T1 in both groups, albeit this was more evident in the EG than in the CG, owing to an interaction between time and group. SRQ score significantly decreased in the EG from T0 to T1, while in the CG it did not, although the interaction between time and group was not significant.

**Conclusion:**

The experimental training program was effective in improving QoL and countering school dropout in young citizens with psychopathic traits. Further studies are needed to clarify if such results are due to a relationship between the practical tasks approach including contact sports and an improvement in mentalization processes.

## 1 INTRODUCTION

Psychopathic personality traits positively predict school dropout [1] and aggressive behavior among adolescents exposed to stressful conditions [2, 3]. Dropping out of high school, psychopathic personality traits and juvenile offences predict a mortality risk in adults due to criminal behavior across the life span, partially in relationship with the developing of Borderline or Antisocial Personality Disorders [4]. Thus, preventing the phenomenon of dropouts from high school among the youth with psychopathic personality traits is a critical issue.

From a therapeutic point of view, the youth can benefit from maintaining a contact with school across time, and this may be due to several reasons. Firstly, preserving a stable relationship with educators and peers, along with the commitment towards educational goals, can slowly create the “secure base” that young people with psychopathic personality traits appear to be lacking [5, 6].

The study about “Attachment” from its inception by John Bowlby dates from four decades ago, and now it invests multiple domains: theoretic research, psycho-biology and clinical application [7]. Personality disorders included into Cluster B of the Diagnostic and Statistical Manual of Mental Disorders have been found to be associated with insecure attachment in the Bowlbyan paradigm [7, 8] and the role of building relationships with a “secure base” have been highlighted as milestones in the therapy of those personality disorders and aggressive behavior in the youth [9].

According to Fonagy and Bateman, the ability to “mentalize” is intimately linked up with attachment style. They define mentalization as the “process by which we implicitly and explicitly interpret the actions of ourselves and others as meaningful based on intentional mental states (*e.g.*, desires, needs, feelings, beliefs, and reasons)” [10].

Attachment style influences mentalization because the insight of adults and the consequent feedback to children about the childhood experience provides a model for children. The feedback from significant adults supports children in paying attention and understanding what they are experiencing, and in focusing on their own feelings [11]. A milestone in children is learning to think about and understand their feelings. Thus, the evolution from “spectator” to active self-observation depends on an effective emotional interaction between children and parental reference figures. It can occur only when the attachment is secure. Secondly, the educational commitment may detach youths with psychopathic traits from high-risk situations and conditions related to street delinquency [12]. Finally, achieving scholastic goals may both increase the likelihood of being competitive in the workplace and decrease the likelihood of social drift.

To achieve these objectives, we structured an innovative educational program, based on a one-year course leading to certification as technicians in Photovoltaic (PV) and other renewable energies. Based on a substantial grant from the European Social Fund, we were able to introduce the following issues in this program:

1. Courses mainly focused on practical tasks, with the theoretical concepts being achieved through a long-lasting experience-driven approach;
2. Contact sports, such as karate and rugby, were a fundamental part of the curriculum;
3. Both individual and group counseling were made available to all the students.

The first item was devised because people with psychopathic traits show difficulty in understanding the causal link between facts and both ideas and emotions [5, 13]. In fact, the impairment in “mentalization” was supposed to “lead to hypersensitivity and increased susceptibility to contagion by other people's mental states, and poor integration of cognitive and affective aspects of mentalization” [14]. This impairment may lead to interpersonal ineffective redundancies, and to dysregulation of mood and impulsivity [14, 15].

It has recently been shown that individuals with psychopathic traits and histories of offensive behavior have specific deficits in social cognition, in envisioning and mentalizing mental states. Male offenders with or without Antisocial Personality Disorder (APD) in a battery of computerized mentalizing tests requiring the taking of perspective (PT Test), recognition of mental states by facial expression (RME Test), and the identification of mental states in a context of social interaction (MASC Test) showed impaired mentalizing in all the tasks when compared with a matched control group without APD or histories of violence. Notably, offenders with APD show greater difficulty in mentalizing than offenders without APD. Mentalization subscales have succeeded in predicting an offender status and those with APD, thus indicating that specific impairments in perspective taking, social cognition, and social sensitivity, as well as tendencies toward hypo-mentalizing and non-mentalizing, are more marked in individuals who meet the criteria for a diagnosis of APD [16].

The data supporting that sports activities may be healthy for maladjusted individuals appear to be contradictory [17]; however, it has been reported that individuals with Antisocial or Borderline Personality Disorders are more prone to practice boxing and other contact sports [18]. This attitude has been tentatively explained by increased self-esteem, but evidence is still lacking [17].

Our group had gained some experience in the use of sport for therapeutic purposes for mental health in clinical [19] and education practice [20]. Thus, the idea of introducing contact sports in the core curriculum is based on the mentalizing deficits in young people with psychopathic traits and on the assumption that their attraction for sports, where a predominant physical component is required, may be explained by the fact that social hierarchies in these contexts are easier to internalize. Starting from this perspective, the project employed this supposed preference to introduce and emphasize in this scenario the need for self-control, group respect and cooperation, being well aware that in these sports physical strength can be used as a means to gain individual leadership and to understand its potential deleterious effects.

The hypothesis is therefore that a training course that facilitates the mentalizing ability in young people with psychopathic traits, also by the practice of a contact sport, may improve perceived Quality of Life (QoL). This healthy outcome is supposed to positively impact on the possibility to conclude a training course of study previously interrupted (and which may be relevant from the viewpoint of prevention).

The construct of QoL includes physical, psychological and social components dealing with subjective wellbeing and general satisfaction about own life [21]. The perceived QoL is considered of relevance as outcome measure to evaluate the efficacy of psychosocial rehabilitation interventions [22, 23].

The main aim of this study was thus to assess both the effects of the selected training program on the perceived QoL and the percentage of school dropouts in a sample of young people with a history of school drop out and psychopathic traits in comparison with a naïve control group with the same psychological features and the same school drop out history.

To verify our hypotheses, we can measure if the proposed curriculum:

1. Would improve QoL and general psychopathology (primary endpoint, health outcomes) in the trained population;
2. Would allow a higher percentage of young people with psychopathic traits to conclude their courses (actual rate being more than 50% dropout), when compared with a population of comparable individuals willing to attend the program but rejected for non-medical reasons (secondary endpoint, educational outcome).

## 2 MATERIALS AND METHODS

### 2.1 Design

Controlled cohort study on young people with a history of school dropout and psychopathic personality traits.

### 2.2 Inclusion Criteria and Recruiting Method

Subjects were recruited through a public notice and with the aid of social services both of the municipalities of several areas of Sardinia (mainly in the South-West part of the island), Italy, and of the Italian Ministry of Justice, which is in charge of custody and alternative measures to imprisonment in the pre-adult population responsible for criminal acts.

The inclusion criteria were:

1. To be over 17 years old;
2. To have dropped out after primary school;
3. To have had a previous criminal conviction or, if not convicted because of reasons linked to age or other, previous reports to social services of antisocial behavior such as bullying, substance trafficking, violence against others or coming from a recovery treatment after addiction.

The exclusion criteria were:

1. To be under 18 and over 25 years old;
2. To have a current drug addiction;
3. To suffer from a current psychotic disorder.

The cohort was made up of those who sent an application form or were sent by social services to participate in the research program, but was discarded for practical reasons (*e.g.*, location difficult to reach or custodial regime not allowing them to attend the training course). These people were placed on a waiting list for a further opportunity for social reintegration.

### 2.3 Sample

The total number of candidates recruited in the training program was 32, and this threshold value was limited by the availability of economic resources. Of the 90 subjects who applied, 75 met the inclusion criteria, but only 69 could assure full/regular participation in the course. Those who applied but did not meet the inclusion criteria, mainly owing to the absence of antisocial traits, were advised of the possibility of alternative courses.

The final selection among the 69 eligible candidates was made by taking into account applications on a first-come, first-served basis. Lastly, the 32 candidates recruited were matched for gender and age (more or less than one year) to an equal number of subjects. Whenever more than one individual that matched for age and gender an experimental one was present in the control group, the pairing was performed randomly.

The two groups were:

- Experimental Group (EG): 32 subjects involved in a one-year-program leading to certification as a technician in photovoltaic and other renewable energies. The course included contact sports (martial arts and rugby) and individual and group counseling.
- Control Group (CG): 32 subjects balanced for gender and age with the experimental group.

(See Table **1** for socio-demographic features)

### 2.4 Tools

The study made use of the following tools:

1. Before the beginning of the study, all candidates who had applied for participation were subjected to a motivational interview conducted by a clinical psychologist. The interview assessed whether the candidates met the criteria and if they could assure regular participation.
2. All the individuals for the EG and the CG underwent a psychiatric interview to exclude current psychotic, anxiety and/or mood disorder. The psychiatrist, after a first step based on a non-structured approach, administered the SCID ANTAS [24], a diagnostic semi-structured interview derived from the SCID-I [25] for the diagnosis of DSM-IV anxiety, mood and eating disorders. None of the selected individuals was excluded for having current psychotic disorders.
3. The Italian version [26] of the Short Form Health Survey (SF-12) [27] was employed to evaluate the perceived Quality of Life (QoL). The perceived QoL is a construct that has become relevant in measuring the wellbeing of individuals and populations as well as the outcomes in diseases with psychological, social and physical impairment [28, 29]. The SF-12 has 12 items and includes the following dimensions: physical activity, physical health limitations on role or activities, emotional state, physical pain, self-evaluation of general state of health, vitality, social activity and mental health. The highest scores correspond to better conditions. All the items referred to the QoL perception in the 30 days before the beginning of the study.
4. The Psychopathy Checklist - Revised (PCL-R), a checklist currently considered the gold standard for the assessment of psychopathy [30]. The tool was developed by Hare and colleagues in the 1980s [31, 32]. The PCL-R is a 20-item instrument derived from the first version. Data on PCL-R have to be obtained through a semi-structured interview [32]. Recent reviews of the literature have found that it is the best predictor of violent behavior currently available [31, 32].
5. The Self Reporting Questionnaire (SRQ) is a questionnaire developed by WHO [33] to measure general psychopathology. The questionnaire consists of 20 closed questions (Yes/No). The SRQ is simple and easy to understand, yet it covers many aspects of psychopathology, including anxiety and mood disorders. The SRQ was translated into Italian by our group and previously used in several settings for research and clinical practice [34].

### 2.5 Intervention

The structure of the PV and other renewable energies technician certification program respects the Italian and European Union rules for technical education courses. The main difference from standard courses, in this case, was that skills and even theoretical concepts were learned through an extremely practical approach. For instance, participants learned how to mount a PV panel rather than studying its functions, which were conversely postponed after the PV panel had become a familiar object. The participants were also supported in their capacity to mentalize, in stimulating active repetitions of the learned concepts in which mentalization was encouraged end underlined when it happened.

During 10 months, the participants benefited both from individual counseling (roughly one meeting every two weeks) and from small-group counseling (once a month on average) both according to the Behavioral - Cognitive Therapy approach [35]. The counseling specifically analyzed feelings and thoughts related to the difficulty of learning, any feelings of aggression toward peers and teachers and tried to show whether these arose in relation to certain conditions (i.e. difficult in learning); to show any feelings of inadequacy (and if they produced external aggression) and to guide the feelings and thoughts of aggression towards the challenge of sport and the development of a medium-term premium in the sense of reasoning to educate themselves to cope with frustration and achieve a target.

The pivotal point of the study was to support the capability to mentalize goal-oriented strategies and support the ability to deal with frustrations on the basis of motivation towards success, since it appears to be crucial in the formation process of secure attachment, and which is thought to be weak and unstable in individuals with psychopathic personality traits [36]. The innovation of this experimental approach consists of the fact that primary attachment is encouraged by course participation itself, practical skills acquired and by the value of what has been learned. Contact sports, such as karate and rugby, were a fundamental part of the curriculum, as above explained, representing roughly one third of the entire training timetable. The sports were played in the afternoon in the grounds of IPSIA. The teachers had graduated in sports science with practical training experience with young people with psychosocial distress. Similarly as for the frequency to other disciplines, were not allowed absences in excess of 20% of the total lessons for the sport disciplines.

### 2.6 Assessment and Statistical Analysis

Both groups were first evaluated at the beginning of the experimental training program (T0) through psychiatric assessment, PCL-R, SF-12 and SRQ, and again at the end of the program (T1) by SF-12 and SRQ. Comparison by “Time” (T0-T1) and “Groups” were carried out by means of a Multivariate Analysis Of Variance (MANOVA).

### 2.7 Ethical Aspects

Researchers provided full information about the nature and purpose of the study to the subjects who also were informed of the possibility of terminating the study at any time. Participants were asked to sign a written informed consent form. The enrolled subjects were given information about data protection and privacy laws. The interviewers explained that the data collected would be used as an anonymous database to maintain confidentiality in accordance with Italian laws on data protection. The study was conducted in accordance with the ethical principles contained in the Helsinki Declaration. The Board of the IPSIA Public Education Institute approved the final study protocol.

## 3 RESULTS

Differences between the two groups are shown in relation to their age, gender and criminal record in Table **1**. As expected on the basis of the chosen inclusion criteria, all subjects in both groups showed psychopathic traits (PCL-R score 21.5±10.4 in the EG; 22.2±9.6 in the CG).

Of the 32 youths enrolled in the EG, 27 (84.4%) completed the course, earned the qualification of PV and other renewable energies technician and underwent the evaluation at T1. All subjects in the CG were evaluated again at T1. Thus, only 5 (15.62%) individuals in the EG were lost (dropped out of the course) during the follow-up; none (0%) was lost at T1 in the CG; the differences between the two groups did not reach statistical significance (χ^2^with Yates correction=3,47, 1 df, p=0.64).

Only 4 (12.5%) individuals in the CG, who were interested in attending a training course, attended such course during the observation year; at the time of re-evaluation this course was still in progress. In a total of 64 young people with previous school leavers, 27 (85%) in the EG were successful in school thanks to the experimental program compared to none (0%) in the CG (χ^2^ correction=46.70, 1 df, p<0.0001). When we consider as the outcome measure both the achievement of academic success and/or the sustaining of their school efforts at the time of the observation, the difference is 27 people (85%) in the EG versus 5 in the CG (15.62%) (χ^2^ correction=30.25, 1 df, p<0.0001).

Table **2** shows the comparison between the two groups' scores on Sf-12 and SRQ as means of Multivariate Analysis of Variance (MANOVA). The SF-12 increased from T0 to T1 in both groups (p<0.0001), albeit this is more evident for the EG than for the CG (p=0.037) and with a significant interaction between “Time” and “Group” (p=0.005). A similar result can also be seen in both sub-scales of the SF-12 that separately analyze physical and psychological dimensions of QoL.

The SRQ mean value increased in the CG, whereas it decreased in the EG. The difference between groups reached statistical significance (p=0.020) and the decrease in the EG from T0 to T1 was statistically significant (F=5.08, df 1, 52.53; p=0.028), even though the interaction between “Time” and “Group” did not reach statistical significance.

## 4 DISCUSSION

The training program achieved the set educational goals. About 85% of the youth who undertook the training program earned the qualification of PV and others of renewable energies technician. Furthermore, the youth participating in the training program improved their perception of the QoL in a statistically significant way when compared to the control group.

In terms of health outcomes, an important result of our study is the significant improvement in the perceived QoL in the experimental sample. Notably, this improvement not only affects physical wellbeing, which is intuitive in view of the practice of sport, but also, and more generally, psychological wellbeing. Conversely, our hypothesis that the course could reduce the psychopathological symptoms in the intervention group was not confirmed. However, the SRQ scores in the experimental group show a statistically significant intragroup increase during the observation period (from T0 to T1), even though there was no significant difference compared to the control group.

To better interpret the results of this research, mainly from a prevention perspective, it is necessary to dwell on the characteristics of the study sample, in order to grasp the psychopathological elements that characterize them. To specify what we meant when we defined our sample as having traits of psychopathy, especially as regards the way in which this dimension was measured, we applied the Psychopathy Checklist-revised (PCL-R), an internationally well-known tool used to assess the presence of psychopathic traits [36]. The PCL-R can produce a dimensional score, or a categorical diagnosis of psychopathy useful in research, forensic medicine and clinical work. The validation work involved dozens of different nations and languages. The tool adopts a trait-based conception broadly in relation to measures of antisocial tendencies as well as psychopathy and has been recently validated by several works. This broad “psychopathy syndrome” should be considered distinct from the DSM-IV and DSM-5 Antisocial Personality Disorder construct because it invests several components of what in these manuals is considered the broad “Cluster B” (specifically narcissistic, antisocial and borderline traits) [37]. The principal factors found by component analysis were [38, 39]: Factor 1, which was found associated with extraversion and positive affects and it appears to identify component traits of psychopathy dealing with interpersonal, social and affective deficits (such as manipulativeness, abuse of shallow charm, lack of empathy). Factor 2, which was found strongly correlated to antisocial personality traits and borderline personality traits and with reactive anger, criminality, and impulsive violence. High scores in PCL-R were found in imprisoned criminals in different countries. The most used cut-off score for the label of a current psychopathy disease is 25 in Europe, while a score of 15 to 25 is indicative of the presence of traits of psychopathy [36]. Alternatively, psychopathy has been classified as “low” by PCL-R score between 10 and 20, “moderate” by PCL-R score between 20 and 30, “high” by PCL-R score >30 [40, 41]. Thus, the sample of this study shows “moderate” psychopathy, or traits of psychopathy.

As underlined in the Introduction, the three characteristics of the youth in our sample (dropping out of high school, psychopathological personality traits and being perpetrators of juvenile offences) are determinants of mortality risk in adults due to criminal behavior across the life span in relationship to the developing of Borderline or Antisocial Personality Disorders [4]. Thus, an educational model able to contrast dropouts from high school in youth with psychopathic personality traits is a relevant tool also from the standpoint of prevention.

Although the study does not show an improvement in general psychopathology in the intervention group, it is known that psychopathological elements linked to psychopathic personality traits show very slow improvement over time [42]. For example, a recent review on the progress of people with traits of borderline personality showed that no treatment used showed a sufficient time span of intervention to allow assessment of improvement in psychopathology and in key life areas [43].

At the same time, the improving of the QoL is noteworthy and it can also be considered a reinforcing element of those of attachment systems (such as sports teams, the class, the new tasks), which may cause continuity in the process of improvement. One of the principal assumptions of the course was to counter the alleged deficits in evolving concepts to link facts and emotions by a method that has its roots in the development of practical skills. A valid approach may be represented by a reduction of formal teaching with pupil-teacher interaction and by stressing the acquisition of practical skills. This is the case if, as was supposed, the deficit in mentalization is indicative of a more important deficit that does not allow the use of a complex strategic approach to problem-solving (involving both emotional empathic intelligence and formal reasoning).

In addition to the sense of leadership, starting from an attitude (the use of physical strength in contact sports) shared by many youth with psychopathic personality traits, and then gradually introducing further concepts such as the importance of group cooperation and the control of their strength, could be an added value. These are very innovative concepts whose theoretical elaborations arise from practical observations.

Furthermore, this experimental training program was part of a broader project, and closely linked to another study (named “A thousand photovoltaic roofs”) [44], whose results have already been published. The overall project had two major social implications. One was to create new job opportunities for citizens with psychosocial disabilities through implementation of specific programs for the benefit of such people (as in the present study), rather than for people with severe psychosocial disabilities (as in the previous one), with work and/or competitive education, to introduce innovative strategies suitable for the local scenario.

Within this context, our group developed the current study, financed by the European Social Fund, with the aim of providing the youth with pathways of deviance, psychopathic personality traits and a history of school dropouts, with specialized and professional knowledge. The previous project, also financed by the European Social Fund, aimed to create a social enterprise involved in the installation of renewable energy systems to provide employment for some of the technicians trained within the current study. Furthermore, several individuals suffering from severe psychosocial disabilities have already been trained and have become workers in the renewable energy industry field as a by-product of the previous project [44].

As regards the latter aim, the overall project was also an effort to give farmers in the area (mainly southern Sardinia), the opportunity to produce energy from renewable sources with low design and installation costs. Indeed, a large part of the southern Sardinian economy is based on the produce of small farms, for which the European Union energy policy may represent a problem, since most of them have neither the means nor the financial resources to comply with the new energy saving and consumption standards.

This approach, focused on guaranteeing better social inclusion for these young citizens, would create a sustainable context not only for future employment but also for students attending this kind of training activities during the course. It should thus strongly act against stigma and represent a problem-solving approach for the farmers and the local community. A significant contribution to the project's success was given by the local collaborative environment, which played an essential role in supporting the students during the whole training period and also after its conclusion, especially the small economic entities where the students carried out their practical training and the companies that employed them, after they had earned their technical qualification.

## CONCLUSION

Our program has been successful in improving the QoL and countering school dropout in young citizens with psychopathic personality traits. The curriculum was strongly focused on practical tasks, with the theoretical concepts being imparted through a long-lasting experience-driven approach and contact sports, such as karate and rugby. Further studies are needed to confirm the results and develop interpretive hypotheses. In fact, given the innovative nature of this project, the theoretical interpretations that we have advanced are to be considered in a heuristic perspective if the results are confirmed.

## Figures and Tables

**Fig. (1):**
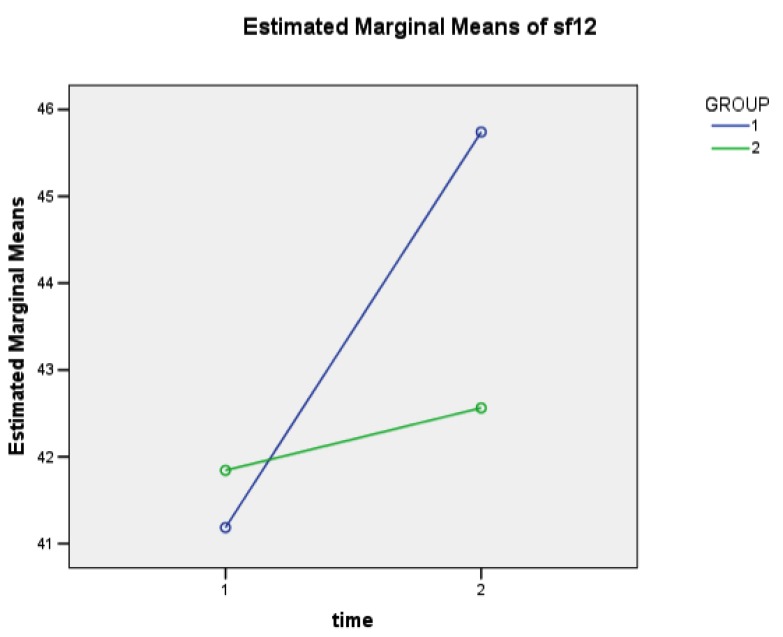
Changes over time on Quality of Life in both groups.

**Fig. (2):**
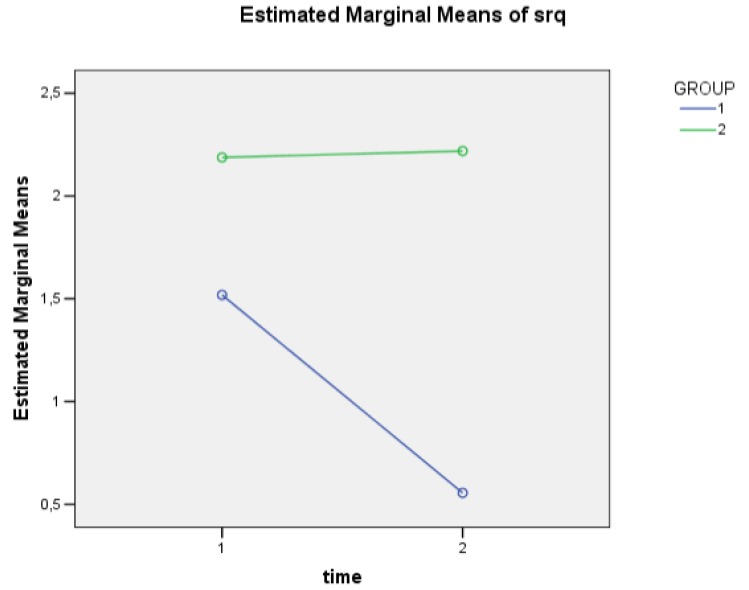
Changes over time on psychopathology in both groups.

**Table 1:** Characteristics of the Groups at T0.

–	**Number** **(male %)**	**Age** **(years)** **mean±sd**	**Jail or Alternative to Jail During Course/ Assessment** **N (%)**	**Previous Criminal Convictions** **N (%)**	**PCL-R** **score** **mean±sd**	**Bipolar** **Disorders** **(lifetime)** **N (%)**	**Major** **Depressive** **Disorder** **(lifetime)** **N (%)**
**Experimental** **Group**	32 (90.62)	19.6±4.3	6 (18.75%)	9 (28.12%)	21.5±10.4	2 (6.25%)	4 (12.5%)
**Control** **Group**	32 (90.62)	19.4±3.8	5 (15.62%)	10 (31.25%)	22.2±9.6	4 (12.5%)	3 (9.37%)
**Statistics for Homogeneity Control**	Matched	Matched	χ^2^=0.11 2df p=0.74	χ^2^=0.08 2df p=0.78	F=0.08 1,62,63 df p=0.78	χ^2^ Yates=0.18 2df p=0.67	χ^2^ Yates=0.01 2df p=0.99

**Table 2:** Multivariate tests: changes over time on Quality of Life and general psychopathology.

**Measure**	**Mean ± sd** **T0 - T1**	**Group**	**Time**	Time * group
**SF-12** **(total score)**	EG (N 27) T0: 42.08±4.069 T1: 45.69±2.205 CG (N 32) T0: 41.84±3.734 T1: 42.56±3.706	F=4.583; df 1,57; p=0.037 PES: 0.136	F=18.754; df=1,57; p=0.000 PES: 0.222	F =8.377; df = 1,57; p=0.005 PES: 0.131
**SF-12** **(physical score)**	EG (N 27) T0: 23.85±3.01 T1: 25.73±3.32 CG (N 32) T0: 23.71±2.92 T1: 24.15±3.01	F=3.978; df 1,57; p=0.043	F=5.013; df=1,57; p=0.024	F =4.221; df = 1,57; p=0.033
**SF-12** **(mental score)**	EG (N 27) T0: 18.19±2.78 T1: 19.96±2.82 CG (N 32) T0: 18.13±2.59 T1: 18.40±2.62	F=5.195; df 1,57; p=0.022	F=9.384; df=1,57; p=0.004	F=5.980; df=1,57; p=0.015
**SRQ**	EG (N 27) T0: 1.52±1.968 T1: 0.56±1.013 CG (N 32) T0: 2.19±2.334 T1: 2.22±2.959	F=5.707; df 1,57; p=0.020 PES: 0.091	F=2.132; df 1,57; p=0.150 PES: 0.036	F=2.427; df 1,57; p=0.125 PES: 0.041

Legend

EG: Experimental Group; CG: Control Group

T0: baseline; T1: post-treatment

PES: Partial Eta Squared (estimate effect size)
